# The Complex Effect of Different Tillage Systems on the Faba Bean Agroecosystem

**DOI:** 10.3390/plants13040513

**Published:** 2024-02-13

**Authors:** Rasa Kimbirauskienė, Aušra Sinkevičienė, Austėja Švereikaitė, Kęstutis Romaneckas

**Affiliations:** Agriculture Academy, Vytautas Magnus University, Studentu Str. 11, 53361 Akademija, Kaunas Distr., Lithuania; rasa.kimbirauskiene@vdu.lt (R.K.); ausra.sinkeviciene@vdu.lt (A.S.); austeja.svereikaite@vdu.lt (A.Š.)

**Keywords:** *Vicia* faba, tillage systems, complex evaluation, CEI

## Abstract

The interactions of the different factors in differently tilled faba bean agroecosystems are still insufficiently studied and evaluated. For these reasons, we studied the results of a long-term field experiment, which was carried out in the Research Station of Vytautas Magnus University, Agriculture Academy (Lithuania). The aim of this study is to comprehensively evaluate the effect of the deep ploughing (DP), shallow ploughing (SP), deep cultivation, chiseling (DC), shallow cultivation-disking (SC), and no-tillage (NT) systems for the faba bean agroecosystem on the complex interactions of the indices, the relations among the indices, and the strength of the impact; the study employs the integrated evaluation method, which uses the complex evaluation index (CEI). CEI values showed that the NT system had a greater effect on the increase of soil aggregate stability (61%), the decrease of CO_2_ emissions (12%), and the increase of seed yield (6%) than the DP system. However, the NT system had 36% and 20% higher effect on weed density and biomass increase than DP. CEI values of the DP system were often minimal, i.e., close to 1, which showed the DP system’s ineffectiveness.

## 1. Introduction

The faba bean is a valuable protein crop used for human food and animal fodder [1]; it is also an important crop rotation plant [2]. When growing faba beans, the soil does not lose fertility because the amount of nitrogen in the soil increases. Faba beans also control the spread of pests, diseases, and weeds [3].

Lithuania, as a country with a temperate climate, is a favorable country for growing faba beans. According to Statistics Lithuania [4], in 2021 Lithuania was 10th in the world in terms of the amount of faba beans cultivated. Faba beans are sensitive to high temperatures and water stress [5,6] and are classified as a moderately demanding temperature group [7]; they are more tolerant to low temperatures during germination and vegetation than other legumes [8]. Today, the area of faba bean cultivation in Europe ranks second in terms of popularity. The EU greening program calls for an increase in the biodiversity of farms in Europe. For this reason, more and more legumes have been grown since 2015 [4].

The most important agronomic feature of faba beans is their ability to form a symbiosis with *Rhizobium* bacteria in the soil [9]. The crops also increase the yield and/or protein content of the crops sown after them; because some of the nitrogen obtained during symbiosis is not used, it enters the soil with the plant residues [10]. Depending on the soil and climatic conditions, faba beans can accumulate up to 400 kg ha^−1^ of nitrogen during the growing season [11]. Faba beans also reduce the problems associated with continuous cropping, such as the depletion of soil organic matter and nitrogen and the degradation of soil structure. So, this plant (*Faba bean* L.) is very useful for the world [12].

It has been proven that crops suffer from excessive density and looseness of the soil [13]. Therefore, tillage and its intensity affect soil properties, such as physical, chemical, and biological properties. Also, the development of the faba bean agroecosystem, production, and quality depend on different tillage methods [14]. Some authors suggest that the fall plowing for faba beans must be deeper than for other crops because their roots have deeper penetration [15]. Deep ploughing can be changed by the ploughless methods, such as shallow tillage, zero tillage, etc. The results showed that deep plowing followed by shallow plowing up to 10 cm increased the number of microorganisms in the soil. Deep plowing creates a regime of air, water, and nutrition in thicker soil layers that is favorable for plants and is maintained longer during drought. In deeply plowed soils, there are fewer weeds, higher porosity levels, and better conditions for crop root penetration [16]. Ploughless tillage creates a soil environment that is suitable for growing crops, leaving plant residues, and reducing tillage intensity [17]. The no-tillage system reduces environmental pollution and wind and water erosion, consumes less energy than other tillage systems, and ensures the sustainable development of agriculture [18,19].

Neither Lithuanian nor foreign scientists have an exact and comprehensive answer to the question of how the faba bean agroecosystem is affected by different tillage methods because sustainable farming depends on many indicators. The indicators that determine the impact of tillage systems (as factors) on the faba bean agroecosystem should be combined into an evaluation system, but it is very difficult to decide which indicator has a greater influence on the soil’s physical, chemical, and biological properties, productivity, and weediness in a faba bean crop, and which indicator has a lower impact. This problem would be solved by a complex evaluation system.

The aim of this study is to comprehensively evaluate the effects of the different faba bean agroecosystem tillage systems on the complex interactions of the indicators, the relations among the indicators, and the strength of the impact.

## 2. Materials and Methods

### 2.1. Site Description

A stationary field experiment has been performed since 1988 at the Experimental Station (54°52′ N, 23°49′ E) of Vytautas Magnus University, Lithuania. Data from 2016 to 2018 were taken. The soil at the experimental site is a silty loam (45.6% sand, 41.7% silt, 12.7% clay) Planosol *(Endohypogleyic-Eutric—Ple-gln-w*). The pH_KCL_ of the soil was 6.4–7.7, the amount of available phosphorus varied from 194 to 384 mg kg^−1^, and potassium varied from 85 to 206 mg kg^−1^. The element variation depended on the long-term (since 1988) soil tillage practices.

Lithuania is in a zone of surplus moisture content, with 600–650 mm per year or 350 mm per vegetative period. The vegetative period lasts about 150–180 days. In the 2016 vegetative season, the air temperature was comparable to that of the long-term average (long-term data since 1974), but in June–September, it was more humid than that of the long-term conditions (Table 1 and Table 2). The 2017 season was colder with average humidity, and 2018 was warmer and very arid.

### 2.2. Experimental Design and Agricultural Practice

Five primary tillage systems were investigated: (1) deep moldboard ploughing (DP, as a control treatment); (2) shallow moldboard ploughing (SP); (3) deep cultivation-chiseling (DC); (4) shallow cultivation-disking (SC); and (5) no-tillage (direct sowing) (NT) (Table 3).

The usual pre-crop of faba bean was winter wheat. The experiment was arranged with four replications for each tillage treatment, and a randomized complete block design (RCBD) was used. The total number of plots was 20. The *brutto* size of the plot was 14 × 9 m; the *netto* was 70 m^2^ (10 × 7 m).

After pre-crop winter wheat harvesting, the experimental plots (except NT) were tilled with a Väderstad Carrier 300-disc harrow (Väderstad AB, Väderstad, Sweden). The soil was ploughed with the Gamega PP-3–43 (Gamega Ltd., Garliava, Lithuania) plough. The soil was chiseled with the KRG-3.6 (Gamega Ltd., Garliava, Lithuania) ridge cultivator. The SC plots were additionally disked with a Väderstad Carrier 300 disk harrow. In spring, the faba bean seedbed was formed using a Laumetris KLG-3.6 cultivator (Laumetris Ltd., Keleriškės village, Kėdainiai reg., Lithuania) (except NT). In all the experimental plots, the faba bean seeds were sown with a Väderstad Rapid 300C Super XL sowing machine and fertilized locally (complex fertilizer 7:16:32, 300 kg ha^−1^). The sowing rate was 200–220 kg of seed per ha (40–45 seeds per m^2^). The sowing depth was 5–6 cm, and the distance between the rows was 25 cm. The “Fuego” variety was sown (Norddeutsche Pflanzenzucht Hans–Georg Lembke KG, Holtsee, Germany). Pests (aphids) and diseases (*Botrytis cinerea*, *B. fabae*) were chemically controlled at the beginning of the crop flowering (BBCH 60–63); the weeds were treated with herbicides once, immediately after sowing.

### 2.3. Methodology

The main indicators characterizing the faba bean agroecosystem are soil aggregate composition and stability; soil pH and the proportions of N, P, K, and Mg; volume of pre-crop residues on the top of the soil; GHG concentration and emissions from the soil related to the soil temperature and moisture content; soil enzymatic activity; and the number and biomass of earthworms. In addition to output, the indicators are the crop density, photosynthesis conditions, development conditions, biometric data, productivity and quality indicators, and crop weediness.

#### 2.3.1. *Methods and Analysis*

***Soil structure and its stability.*** The samples were taken using a shovel before the spring tillage and after the bean harvest from at least five spots per plot. The analyzed layers were 0–15 and 15–25 cm. Sample means were drawn. A Retsch sieving machine (Retsch Lab Equipment, VERDER Group, Vleuten, The Netherlands) and a set of sieves were used to determine soil structure. It was arranged according to the mesh size, as follows: 10.0 mm, 7.1 mm, 5.6 mm, 4.0 mm, 2.0 mm, 1.0 mm, 0.5 mm, and 0.25 mm. The stability of the soil aggregates was determined by wet sieving with the Retsch device, using only the previously dry-sieved soil fraction of 1–2 mm [20,21]. 

***Soil agrochemical properties*.** Soil samples were collected from at least 10 to 15 spots per experimental plot. A sample mean was drawn, and laboratory analyses were used to determine the levels of the main macronutrients (N, P, K, Mg) and soil pH. The analyses were carried out at the certified Agrochemical Research Laboratory of the Lithuanian Research Centre for Agriculture and Forestry following standardized methods.

***Covering soil with crop residues*.** The distribution of the residues of the previous winter wheat crop on the soil surface before and after sowing was determined by visual inspection. The visual method used a 10 m long metal tape, which was stretched perpendicularly to the sowing direction at two points and diagonally across the sowing rows in each plot. The points of contact with the plant residues were set at 10 cm intervals (100 spots) for a total of 200 points per plot or 4000 spots per experiment [20].

***CO_2_ concentration and emissions.*** The flux and the concentration of the CO_2_ emissions above the soil surface were determined by the closed-chamber method using a portable infrared analyzer LiCor–6400 (LI-COR Inc., Lincoln, NE, USA). The IRGA technique was used. The LI–8100A portable soil respirator system with an 8100–103 camera was used (LI-COR Inc., USA). A 20 cm diameter ring was hammered into each experimental plot in spring, and three measurements were taken. The measurements were taken three times: at the beginning, in the middle, and at the end of the plant growing season.

***Soil temperature and moisture content.*** This was performed at least five locations in each experimental plot. The basis of the HH2 moisture meter (Delta-T-Devices) was used—the WET sensor. The WET sensor directly measures soil moisture and temperature. It is used for accurate soil and artificial substrate testing up to a depth of 10 cm.

***Soil saccharase activity.*** The samples were taken annually after the bean harvest, together with the soil structure samples, and were dried in the laboratory at 20–22 °C. The analyzed layer was 0–15 cm deep. The activity of the soil enzyme saccharase was analyzed according to the Hofmann and Seegerer [22] method, modified by A. I. Chunderova [20,23].

***Numbers and mass of earthworms*.** This was determined after the bean harvest. The earthworm abundance was determined at three spots per experimental plot. The study was based on the use of a formalin solution. A 0.5 × 0.5 m metal frame was driven into the ground and used for the study. A 0.55% formalin solution (at least 10 L) was prepared and poured onto the soil area separated by the frame. After absorption of the solution, the earthworms appearing on the soil surface were collected, counted, and weighed.

***Crop density*.** At the beginning of growth, 10 spots of the experimental plot in a 1 m continuous row were assessed on day 3 and day 10 from the start of germination. At the end of the growing season, the faba bean crop density was evaluated at the same time as the productivity of the crop. We calculated faba bean plants per samples and recalculated to the square meter.

***Photosynthetically active radiation (PAR).*** This was determined at the beginning of faba bean flowering (BBCH 60–63). Photosynthetically active radiation (PAR) was measured with a radiometer HD 9021 RAD/PAR (PAR E m^−2^, 400–700 nm wavelength). PAR was measured at different crop layers: on the soil surface, at ^1^/_2_ bean crop height, and above the crop (background). The measurements were taken from at least 5 spots in the experimental plot. The indicator is expressed as a percentage of the background irradiation.

***Indicators of crop development (chlorophyl index and assimilation area of leaves, height of canopy, biomass)*.** These were determined at the beginning of faba bean flowering (BBCH 60–63). Ten faba bean plants were cut in each experimental plot for the study. The height of each plant was measured and weighed to determine its green biomass. The biomass samples were dried in a thermostat at 105 °C to a constant weight. Thus, the dry biomass of the plants was determined. The assimilation area of the faba bean leaves (cm^2^) was measured with a Win Dias leaf area measuring device (Delta–T Devices Ltd., Burwell, UK). The leaf chlorophyll index was also measured with a CCM–200 plus chlorophyll content meter (OPTI–SCIENCES).

***Weed density and biomass*.** This was determined by assessing the weed species composition, the number of weeds at the beginning and at the end of the growing season, and the amount of dry matter at the end of the bean growing season. The crop weediness was determined in at least 10 spots of the experimental plot within the 0.06 m^2^ area. The weed seedlings (pcs m^−2^) were counted at the beginning of the growing season, and the number of weeds (pcs m^−2^) and the amount of dry matter (g m^−2^) were determined at the end of the growing season. The weeds were uprooted and dried to an air-dried weight, and a botanical names the species were specified [21,24].

***Crop biometric, productivity, and quality parameters at harvesting*.** The samples for these parameters were taken from at least five spots in the experimental plot, in a longitudinal row of 0.5 m. A sample mean was drawn. A total of 20 samples were analyzed. The average height of the faba bean plant, the green and dry biomass of the sample, the average crop density, the number of pods per plant, the bean seed yield (at a 15% moisture content), the 1000-seed weight, and the average number of seeds per pod were determined. The protein content of the faba bean seeds was determined at the Agrochemical Research Laboratory of LRCAF Method Directive 72/199/EEC [20,21].

The variation in the indices (variables) observed above and the units of measurements are presented in Table 4.

#### 2.3.2. Statistical Analysis and Calculations

A comprehensive evaluation of the faba bean agroecosystem was carried out based on the methodologies of G. Lohmann [25] and K. U. Heyland [26]. The following studies and mathematical calculations were carried out: (1) the values of the different indicators were determined; (2) the real values of each indicator were converted to a uniform nine-point scale. A score of 1 corresponds to the worst or minimum value and 9 to the best or highest value. For all the other values of the same indicator, the scores were calculated according to the following formula:

*VB_i_* = (*X_i_* − *X_min_*)/(*X_max_* − *X_min_*)^−1^ × 8 + 1 (1)

where *VB_i_* is the score for a value of a given indicator, *X_i_* is the expression for a given value, *X_max_* is the maximum value for a given indicator, and *X_min_* is the minimum value for a given indicator. (3) The indicators converted to scores are shown in grid diagrams with a radius from 1 to 9; (4) the scale also shows the average value of the individual indicators—the score threshold—which is equal to five points and which distinguishes between the high and the low scores. The effectiveness of the measurement is indicated by the area bounded by the scores of all its indicators. (5) The calculation of the complex evaluation index (CEI), which consists of the average of the evaluation scores, the standard deviation of the evaluation scores, and the standard deviation of the average of the evaluation scores below the evaluation threshold was carried out [27]. An example of the CEI calculations is shown in Supplementary Materials, Table S1.

## 3. Results and Discussion

**Figure 1:**
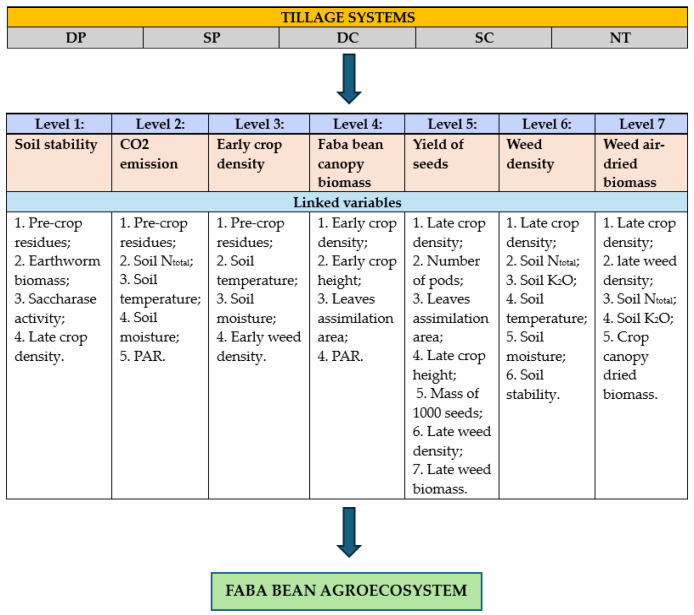
Differently tilled faba bean agroecosystem complex evaluation model, divided into separate levels.

In our earlier studies, we found that the most important and influential indices, which are the “key” parameters used to evaluate tillage systems, are: the soil water stability, CO_2_ emissions, faba bean crop, faba bean canopy biomass, yield of the faba bean seeds, and weed air-dried biomass. These “key” indicators, which represent the faba bean agroecosystem levels, also interact with other indices of the system. We conducted an integrated assessment of the faba bean agroecosystem based on the system levels description and the inner interactions. In Figure 1, a complex faba bean agroecosystem evaluation model is presented.

### 3.1. Level 1: Soil Aggregate Stability

Soil performs several functions at the same time, and the soil-forming factors and the physical, chemical, and biological properties of soil determine the degree of functionality of each function [28,29]. Long-term intensive ploughing reduces soil aggregate stability [30], increases soil bulk density [31,32], and deteriorates the soil biological properties [33,34].

It is very difficult to decide which indicator has a greater impact on the crop agroecosystem and which one has a lesser impact. Therefore, a comprehensive assessment of soil quality is needed to describe the soil’s ability to function by integrating the soil chemical, physical, and biological components, which are highly sensitive to the management decisions of land users [35].

**Figure 2:**
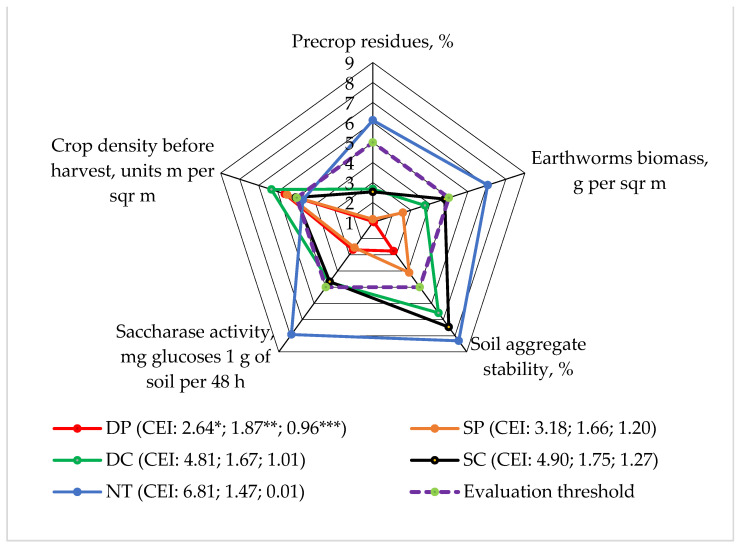
The effects of different tillage methods on the faba bean agroecosystem in terms of soil aggregate stability for water (Level 1) interactions with other indicators, 2016–2018. Note: DP—deep ploughing at 22–25 cm depth (control treatment); SP—shallow ploughing at 12–15 cm depth; DC—deep cultivation at 25–30 cm depth; SC—shallow cultivation at 10–12 cm depth; NT—not-tilled soil (direct sowing). CEI—complex evaluation index, *—average of evaluation points (EPs), **—standard deviation of EPs, ***—standard deviation of the average of the EPs below the evaluation threshold.

In our experiment, the stability of the structure of the surface layer of the soil, both at the beginning and at the end of the vegetative season, was mostly determined by the differences in the amount of pre-crop (winter wheat) residues before tillage in spring (r = 0.897 and 0.906; *p* < 0.05). A relationship between earthworm biomass (r = 0.902; *p* < 0.05) and structure stability was also established [20]. The other indices included in Figure 2 had less influence.

From 2016–2018, when assessing the stability of the soil structure in the 0–15 cm soil layer, the deep tillage (DP), shallow tillage (SC), and direct seeding (NT) systems were superior to the others; their results rose above the five-point evaluation threshold (Figure 2). The same results were found by Sinkevičius [36]. He states that the highest score for soil aggregate stability was found in the no-tillage and no-catch crop technology. In our experiment, the no-tillage system had an influence on the pre-crop cover, earthworm biomass, and saccharase activity scores, which rose above the evaluation limit (five points). The crop density scores below the assessment threshold were determined using the NT system.

The calculated complex evaluation indicators (CEI) consisted of the average of all the evaluation scores (EPs), the standard deviation and EPs not exceeding the evaluation limit, the standard deviation, and the areas limited by the evaluation scores. This shows that direct sowing (NT) in the faba bean agroecosystem has the greatest impact on the agroecosystem at Level 1.

### 3.2. Level 2: CO_2_ Emission

Another important endogenous factor acting in the faba bean agroecosystem was CO_2_ gas emission. As much as 57% of all greenhouse gases are attributed to CO_2_. About 20% of the total amount of CO_2_ is released into the atmosphere by soils, so soils have a considerable influence on the CO_2_ emission balance [37]. Currently, 12–15%, or 5.1–6.1 Gt CO_2-eq._ m^−1^, of global greenhouse gases are generated by agriculture (9% in the EU).

In our experiment, it was found that CO_2_ emission in the faba bean agroecosystem mostly varied during the months of June–August and was more correlated with soil temperature (r = 0.8; *p* > 0.05) and soil moisture content (r = 0.6; *p* > 0.05).

**Figure 3:**
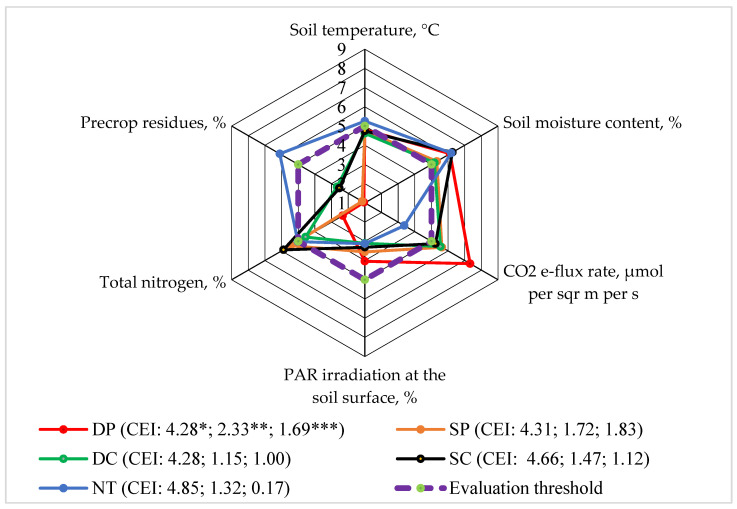
The effects of different tillage systems on the faba bean agroecosystem in terms of soil CO_2_ emission (Level 2) interactions with other indicators, 2016–2018. Note: DP—deep ploughing at 22–25 cm depth (control treatment); SP—shallow ploughing at 12–15 cm depth; DC—deep cultivation at 25–30 cm depth; SC—shallow cultivation at 10–12 cm depth; NT—not-tilled soil (direct sowing). CEI—complex evaluation index, *—average of evaluation points (EPs), **—standard deviation of EPs, ***—standard deviation of the average of the evaluation points below the evaluation threshold.

When evaluating the release of CO_2_ emission from the soil following the use of the different applied tillage systems, it was found that only NT was rated lower than five points (Figure 3). The highest evaluation score was determined for the DP system. Rudinckienė [27] claimed that the CO_2_ emission scores from the soil, when growing multifunctional crops and applying different technologies, were determined to be higher than the assessment limit. In our experiment, for soil temperature (0–15 cm soil layer) and the pre-crop residue cover, the evaluation scores rose above the evaluation threshold only in the NT system. For all the tillage technologies, after determining the soil moisture (0–15 cm in the soil layer, in the middle of the vegetation), the evaluation scores were evenly distributed. All the assessment scores were set above the assessment threshold. After determining the photosynthetically active radiation (PAR) on the soil surface, the scores for the application of all the tillage systems were found to be lower than the evaluation limit. In the upper (0–15 cm) soil layer, the total nitrogen assessment scores rose above the assessment limit only with the SP, SC, and NT systems.

The calculated indicators of the complex evaluation and the areas limited by the evaluation points showed that the impact of the tillage systems on the Level 2 agroecosystem was different. The most highlighted effect was that of the DP system on the CO_2_ e-flux rate.

### 3.3. Level 3: Faba Bean Crop Density

Tillage is the most common agricultural practice; it creates a suitable environment for seed germination by promoting soil warming and water evaporation [38] and controlling weed infestation.

**Figure 4:**
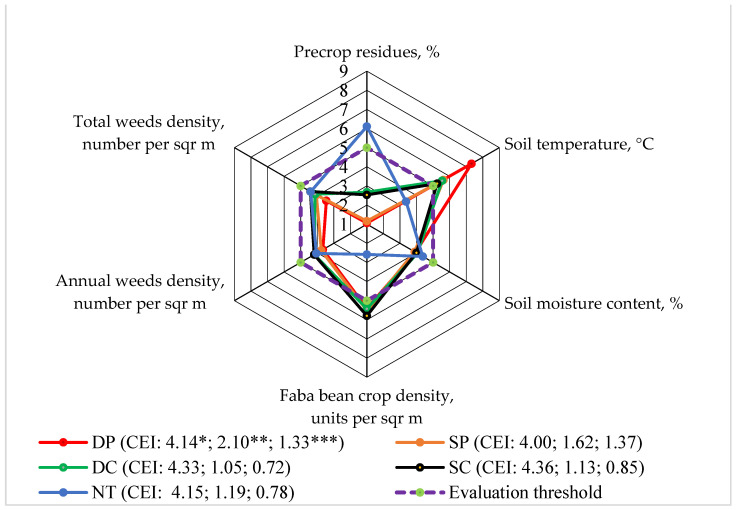
The effects of different tillage systems on the faba bean agroecosystem in terms of crop density (Level 3) interactions with other indicators, 2016–2018. Note: DP—deep ploughing at 22–25 cm depth (control treatment); SP—shallow ploughing at 12–15 cm depth; DC—deep cultivation at 25–30 cm depth; SC—shallow cultivation at 10–12 cm depth; NT—not-tilled soil (direct sowing). CEI—complex evaluation index, *—average of evaluation points (EPs), **—standard deviation of EPs, ***—standard deviation of the average of the evaluation points below the evaluation threshold.

The correlation–regression analysis showed that the faba bean crop density at the beginning of the growing season was one of the main indices and that it depended on the faba bean agroecosystem. We performed an integrated evaluation to determine the interactions among faba bean crop density at the beginning of the growing season and the other research factors. The density of the bean crop was calculated on the 10th day from the beginning of faba bean germination (Figure 4). The density of the crop largely depended on the amount of pre-crop residues on the soil surface. This influence was particularly significant in 2017, not only at the beginning of the faba bean vegetative period (r = −0.819, *p* > 0.05), but also during harvesting (r = −0.824, *p* > 0.05) [20].

Faba bean crop density and soil temperature (at the beginning of vegetation in the 0–15 cm soil layer) lower than the evaluation limit were determined only when applying direct sowing (NT) (Figure 4). In the evaluation of soil moisture in the 0–15 cm soil layer at the beginning of vegetation, the number of annual weeds, and the total weeds at the beginning of vegetation, the evaluation scores did not increase more than the evaluation limit when applying the different tillage systems. The highest score of the pre-crop cover after sowing was determined by applying the NT system.

The calculated indicators of the complex evaluation and the areas limited by the evaluation scores showed that the impact of NT on the agroecosystem was mainly greater than that of the other tested systems.

### 3.4. Level 4: Faba Bean Canopy Biomass

**Figure 5:**
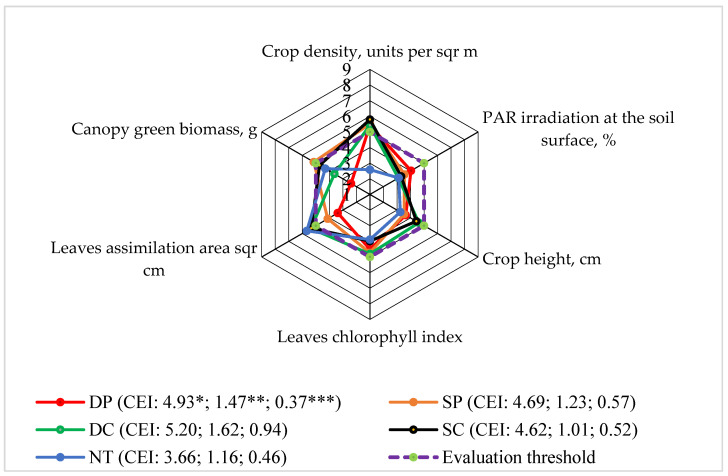
The effects of different tillage systems on the faba bean agroecosystem in terms of faba bean canopy biomass (Level 4) interactions with other indicators, 2016–2018. Note: DP—deep ploughing at 22–25 cm depth (control treatment); SP—shallow ploughing at 12–15 cm depth; DC—deep cultivation at 25–30 cm depth; SC—shallow cultivation at 10–12 cm depth; NT—not-tilled soil (direct sowing). CEI—complex evaluation index, *—average of evaluation points (EPs), **—standard deviation of EPs, ***—standard deviation of the average of the evaluation points below the evaluation threshold.

Among the different soil tillage methods, the average fresh biomass of the faba bean canopy was mainly not significantly different; so, the evaluation scores of all the applied different systems did not reach the evaluation limit when studying the fresh biomass of the crop, crop height, leaf chlorophyll index, and photosynthetically active radiation (PAR) on the soil surface (Figure 4). Only NT did not increase the faba bean crop density at the beginning of the vegetative period above the evaluation limit (five points) scores. The scores of the leaf assimilation area above the evaluation limit rose the most with NT (Figure 5).

The evaluation scores of the various indices were calculated; their limited area and the integrated evaluation indices (CEI) were determined and consisted of the average of all the EPs, the standard deviation and the EPs that did not exceed the evaluation limit and standard deviation. This shows that different tillage systems did not differ among themselves in terms of efficiency.

### 3.5. Level 5: Yield of Faba Bean Seeds

The yield of faba bean seeds depended more on the meteorological conditions of the vegetative season than on the applied tillage systems. Seed productivity was also influenced by crop density (r_2016_ = 0.946; *p* < 0.05) and the number of pods per square meter (r_2016_ = 0.950; *p* < 0.05), as well as the assimilation area of the faba bean plant leaves (r_2017_ = 0.887, *p* < 0.05), the plant height (r_2017_ = 0.712; *p* > 0.05), and the mass of 1000 seeds (r_2018_ = 0.916; *p* < 0.05).

**Figure 6:**
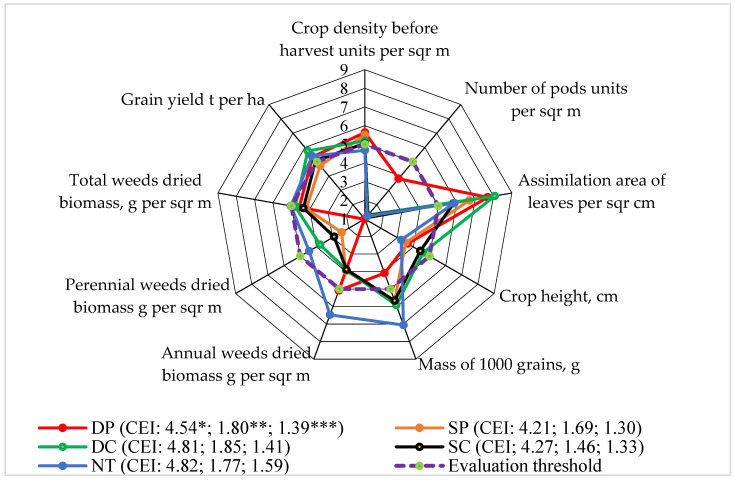
The effects of different tillage systems on the faba bean agroecosystem in terms of faba bean seed yield (Level 5) interactions with other indicators, 2016–2018. Note: DP—deep ploughing at 22–25 cm depth (control treatment); SP—shallow ploughing at 12–15 cm depth; DC—deep cultivation at 25–30 cm depth; SC—shallow cultivation at 10–12 cm depth; NT—not-tilled soil (direct sowing). CEI—complex evaluation index, *—average of evaluation points (EPs), **—standard deviation of EPs, ***—standard deviation of the average of the evaluation points below the evaluation threshold.

In the evaluation of the yield of the seeds, the evaluation scores did not reach the limit in the SP plots (Figure 6). The pre-harvest crop density score was found to be the lowest in the NT system. In the examination of the assimilation area of the crop leaves, the evaluation scores of all the different applied systems were determined to be higher than the evaluation limit. After analyzing the air-dried biomass of the perennial weeds at the end of the growing season, the number of faba bean pods, and the plant height, it was found that the scores did not rise above the assessment limit when applying all the studied tillage systems.

Sinkevičienė [39] found opposite results. She claimed that by using different mulching technologies, the assessment scores for the number of perennial weeds rose above the assessment threshold. Also, Rudinskienė [27] states that the evaluation scores for the competition with weeds, in the growing of single, double, and ternary crops, are higher than the evaluation limit. In our experiment, the highest scores of the 1000-seed mass and the annual and total air-dried weed biomass at the end of the vegetation season were determined by direct seeding technology (NT). The calculated indicators of the complex assessment and the areas delimited by the assessment scores showed that NT had the highest impact (on the five indices from nine) on Level 5 in the agroecosystem.

### 3.6. Level 6: Weed Density

In our experiment, the number of weeds at the beginning of the faba bean vegetative season (faba bean BBCH 25–27) was highest in the NT plots. At the end of the growing season, the differences between the tillage systems were evened out because the plots were sprayed with herbicides [21].

**Figure 7:**
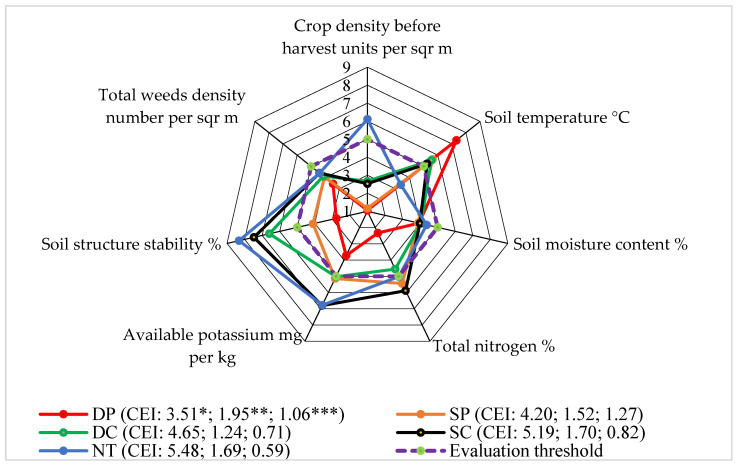
The effects of different tillage systems on the faba bean agroecosystem in terms of weed density (Level 6) interactions with other indicators, 2016–2018. Note: DP—deep ploughing at 22–25 cm depth (control treatment); SP—shallow ploughing at 12–15 cm depth; DC—deep cultivation at 25–30 cm depth; SC—shallow cultivation at 10–12 cm depth; NT—not-tilled soil (direct sowing). CEI—complex evaluation index, *—average of evaluation points (EPs), **—standard deviation of EPs, ***—standard deviation of the average of the evaluation points below the evaluation threshold.

The scores of the total number of weeds at the beginning of the growing season and the soil moisture at the beginning of the growing season in the 0–15 cm soil layer did not rise above the assessment (five points) limit (Figure 7). The highest soil temperature at the beginning of the growing season in the 0–15 cm soil layer was determined using the DP system. However, after evaluating the DP system, the amount of total nitrogen at the beginning of the vegetative season in the 0–15 cm soil layer, the amount of available potassium at the beginning of the vegetative season in the 0–15 cm soil layer, the stability of the soil structure at the beginning of the vegetative season in the 0–15 cm soil layer, and the plant pre-crop cover after the faba bean sowing, the evaluation scores were established as the lowest.

The calculated indicators of the complex assessment and the areas delimited by the assessment scores showed that the impact of SC and NT on the Level 6 of the faba bean agroecosystem was higher than that of the other applied systems. It means that the mentioned systems initiated higher weed density.

### 3.7. Level 7: Air-Dried Weed Biomass

In 2016 at the end of the faba bean growing season (BBCH 75–79), the air-dried weed biomass was the highest. The total weed biomass was 25% higher in the NT plots than in DP and 42% higher than in SP. The DP plots tended to have the lowest total number of all the observed weed species. The annual weeds predominated; therefore, their influence on the total number of weeds was significant (r = 0.946, *p* < 0.05) [21].

**Figure 8:**
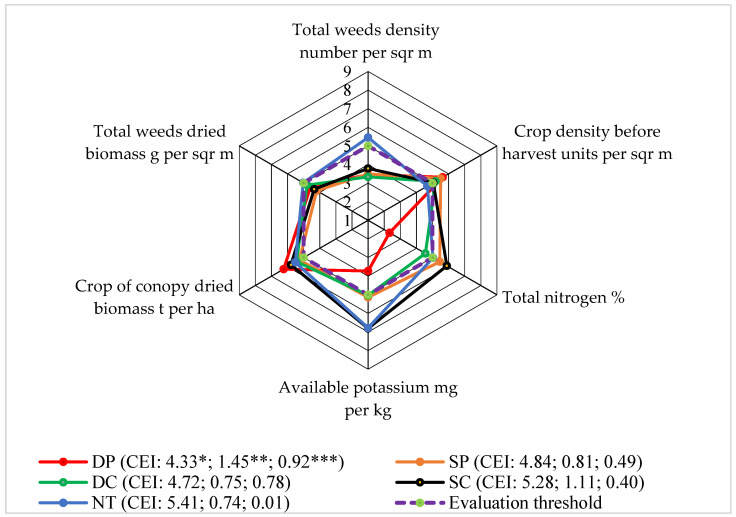
The effects of different tillage systems on the faba bean agroecosystem in terms of air-dried weed biomass at the end of faba bean vegetative season (BBCH 75–79) (Level 7), 2016–2018. Note: DP—deep ploughing at 22–25 cm depth (control treatment); SP—shallow ploughing at 12–15 cm depth; DC—deep cultivation at 25–30 cm depth; SC—shallow cultivation at 10–12 cm depth; NT—not-tilled soil (direct sowing). CEI—complex evaluation index, *—average of evaluation points (EPs), **—standard deviation of EPs, ***—standard deviation of the average of the evaluation points below the evaluation threshold.

When evaluating the total air-dried weed biomass at the end of the vegetative season and the total number of weeds, the highest evaluation scores were determined in the NT plots (Figure 8).

After determining the density of the faba bean crop before harvesting, the opposite trends in the application of the mentioned system were evaluated. When applying different tillage systems, it was found that the evaluation scores of the dried biomass of the canopy of faba beans were higher than the evaluation limit. After the evaluation scores of the SP, SC, and NT systems, total nitrogen, and available potassium at the beginning of the vegetative season in the 0–15 cm soil layer were higher than those of the other comparative systems. Similar results were found by Sinkevičius [36], who claimed that no-tillage and no-catch crop technologies had the effect of increasing the total nitrogen scores more than the assessment limit (5 points).

The calculated indicators of the complex evaluation and the areas limited by the evaluation scores showed that the impact of NT on the Level 7 of the faba bean agroecosystem was one of the highest compared to that of the other tested systems. So, NT initiated weed biomass increase.

Our study showed that by using a complex assessment of the effect of different tillage systems on the faba bean agroecosystem with separate levels (L1–L7) of research, it is possible to assess the agricultural practices used in a more detailed and comprehensive way, not only in a technological sense but also in an environmental sense. The level of complex assessment can be selected according to the main direction of research. For example, when examining GHG emission issues, the Level 2 CO_2_ emission model should be used. When examining soil fertility and safety issues, the Level 1 soil aggregate stability model should be used.

## 4. Conclusions

The complex assessment of the faba bean agroecosystem based on endogenous factors such as structure durability, CO_2_ emissions from the soil, grain yield, and weed biomass determined that the NT system has a greater positive impact on the agroecosystem than the other comparative systems. By evaluating the density of the faba bean crop at the beginning of the growing season and the green biomass of the faba bean canopy, it was found that the different tillage systems did not differ in their efficiency when compared to each other. When evaluating the agroecosystem based on the number of weeds (BBCH 25–27), the calculated indicators of the complex evaluation and the areas limited by the evaluation scores showed that the impact of SC and NT on the faba bean agroecosystem was greater than that of the other applied systems.

The various values of the calculated evaluation scores, their limited areas, and the determined complex evaluation indices showed that the influence of the NT system on the faba bean agroecosystem was greater than that of the other systems used. The SC system was more efficient than the DP, SP, and DC systems. In the DP system, the calculated CEI values were more minimal, i.e., close to 1, which showed the DP system’s ineffectiveness.

The complex assessment of agroecosystems is an important tool for highlighting the influence of system components (variables and indicators) on the individual system levels, through which the entire agroecosystem is affected. Therefore, it should be applied next to a correlation–regression analysis of the results.

## Figures and Tables

**Table 1:** The average air temperature (°C) during the faba bean vegetative period. Kaunas Meteorological Station.

Month/Year	2016	2017	2018	Long-Term Average
April	7.4	5.6	10.2	6.9
May	15.7	12.9	17.2	13.2
June	17.2	15.4	17.5	16.1
July	17.9	16.8	20.1	18.7
August	16.9	17.5	19.2	17.3
September	-	13.4	-	12.6

**Table 2:** The precipitation rate (mm) during the faba bean vegetative period. Kaunas Meteorological Station.

Month/Year	2016	2017	2018	Long-Term Average
April	41.2	73.7	64.8	41.3
May	36.4	10.2	17.6	61.7
June	83.9	80.2	57.6	76.9
July	162.9	79.6	137.5	96.6
August	114.9	55.0	66.2	88.9
September	-	87.1	-	60.0

**Table 3:** Description of tillage practices (according to Romaneckas et al. [20]).

Tillage System	Stubble Tillage	Primary Tillage	Implement	Depth of Tillage cm	Pre-Crop Residue Cover %
Deep ploughing	Yes	Inversion	Moldboard plough	22–25	0–3
Shallow ploughing	Yes	Inversion	Moldboard plough	12–15	2–4
Deep cultivation	Yes	Non-inversion	Chisel cultivator	25–30	40–51
Shallow cultivation	Yes, twice	Non-inversion	Disc harrow	10–12	40–50
No-tillage	No	No	None	0	47–87

**Table 4:** Tested indices and their variation.

Indices	Variation	Units	Indices	Variation	Units
Pre-crop residues	0.3–82.8	%	PAR at the soil surface	0.5–23.6	%
Soil aggregate stability (at the beginning of vegetative season)	35.9–72.7	%	Crop height in the middle of vegetative season	50.1–101.0	cm
Total nitrogen (at the beginning of vegetative season)	0.11–0.17	%	Leaf chlorophyll index	23.20–47.0	–
Available potassium (at the beginning of vegetative season)	85.0–181.0	mg kg^–1^	Leaf assimilation area	337.20–1270.9	cm^2^
Earthworm biomass	23.5–134.7	g m^–2^	Faba bean plant average canopy green biomass	45.40–106.30	g
Saccharase activity	19.7–50.0	mg glucoses 1 g of soil per 48 h	Total weed density (at the beginning of vegetative season)	25.0–246.20	number m^–2^
Soil temperature (at the beginning of vegetative season)	18.8–21.5	°C	Total weed density (at the end of vegetative season)	18.8–80.8	number m^–2^
Soil moisture content (at the beginning of vegetative season)	11.3–16.2	%	Total weed biomass (at the end of vegetative season)	30.8–219.2	g m^–2^
Soil temperature (in the middle of vegetative season)	14.7–18.9	°C	Number of pods	207.6–522.0	m^2^
Soil moisture content (in the middle of vegetative season)	15.0–18.7	%	Mass of 1000 grains	483.70–610.17	g
CO_2_ e–flux rate (in the middle of vegetative season)	3.07–7.66	µmol m^–2^ s^–1^	Grain yield	2.19–5.92	t ha^–1^
Early faba bean crop density	19.9–46.0	units m^–2^	Canopy dried biomass at harvest	6.56–12.81	t ha^–1^
Crop density before harvest	32.4–55.6	units m^–2^			

## Data Availability

Not applicable.

## Supplementary Materials

The following supporting information can be downloaded at: https://www.mdpi.com/article/10.3390/plants13040513/s1. Table S1: Example of CEI calculations for Level 1: Soil aggregate stability, divided into the stages.
