# Supplementary material for: The Complex Effect of Different Tillage Systems on the Faba Bean Agroecosystem
Source: Plants (Basel). 2024 Feb 13;13(4):513. doi: 10.3390/plants13040513 (PMC10892685; doi:10.3390/plants13040513)
Supplement: Supplementary file 1 [file plants-13-00513-s001.zip › plants-2830512-supplementary.pdf]

**Table S1.** Example of CEI calculations of Level 1: Soil aggregate stability, divided into the stages

<table>
<tr><th>Stage</th><th>Example</th></tr>
<tr><td>1) The values of the different indicators are determined (for example, volume precrop residues). Exell platform was used.</td><td>
1) The values of the different indicators were determined
<table>
<tr><td></td><td></td><td colspan="3">I Soil aggregate stability</td><td></td><td></td></tr>
<tr><td></td><td>Treatmens</td><td>DP</td><td>SP</td><td>DC</td><td>SC</td><td>NT</td></tr>
<tr><td></td><td>Year</td><td></td><td></td><td></td><td></td><td></td></tr>
<tr><td>Precrop residues (after sowing), %</td><td>2016</td><td>0,5</td><td>0,3</td><td>8,5</td><td>10,5</td><td>82,8</td></tr>
<tr><td></td><td>2017</td><td>1,3</td><td>1,3</td><td>7,3</td><td>11,8</td><td>22</td></tr>
<tr><td></td><td>2018</td><td>0,8</td><td>4,2</td><td>36,8</td><td>25,8</td><td>54,2</td></tr>
</table>
</td></tr>
<tr><td>2) The real values of each indicator are converted to a uniform 9-point scale. A score of 1 corresponds to the worst or minimum value, and 9 – to the best or highest value. For all other values of the same indicator, the scores are calculated according to the following formula: $VB_i=(X_i-X_{min}) / (X_{max}- X_{min})^{-1}\times 8+1$ where: VBi is the score for a value of a given indicator, Xi is the expression for a given value, Xmax is the maximum value for a given indicator, Xmin is the minimum value for a given indicator. Exell platform was used.</td><td>
=(0,5-0,3)/(82,8-0,3)*8+1
<table>
<tr><td>D</td><td>E</td><td>F</td><td>G</td><td>H</td><td>I</td><td>J</td><td>K</td><td>L</td></tr>
<tr><td colspan="9">2) The real values of each indicator were converted to a uniform 9-point scale</td></tr>
<tr><td>Formula</td><td>1,02</td><td>1</td><td>1,8</td><td>2</td><td>9</td><td></td><td></td><td></td></tr>
<tr><td></td><td>1,1</td><td>1,1</td><td>1,68</td><td>2,12</td><td>3,1</td><td></td><td></td><td></td></tr>
<tr><td></td><td>1,05</td><td>1,38</td><td>4,54</td><td>3,47</td><td>6,23</td><td></td><td></td><td></td></tr>
<tr><td>Average</td><td>1,06</td><td>1,16</td><td>2,67</td><td>2,53</td><td>6,11</td><td></td><td></td><td></td></tr>
</table>
</td></tr>
</table>

3) The indicators converted to scores are shown in grid diagrams with a radius from 1 to 9.
Exell platform was used.

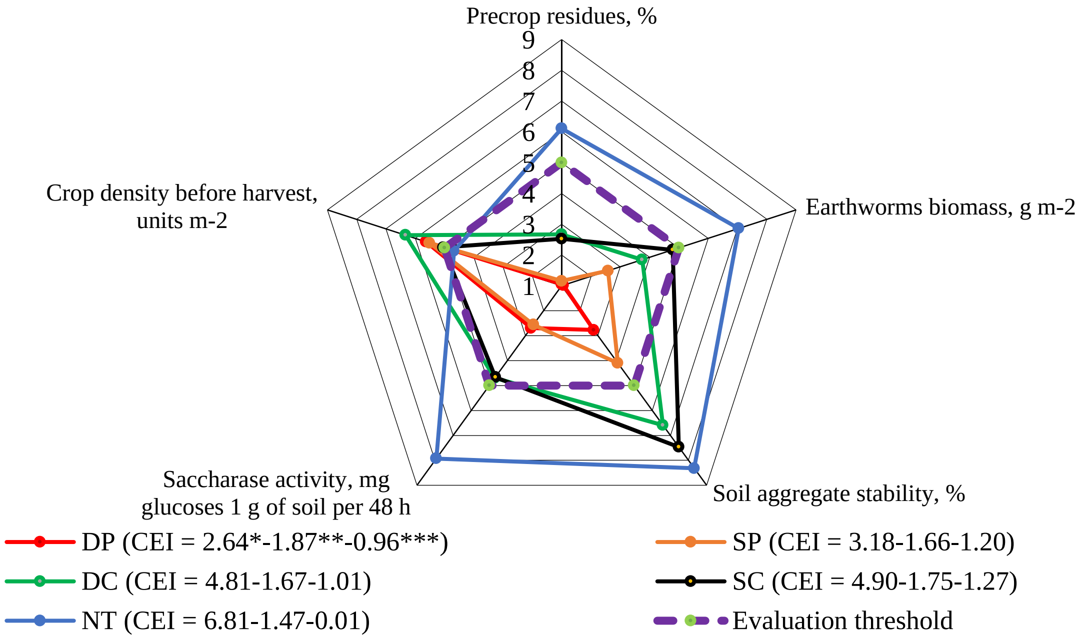


4) The scale also shows the average value of the individual indicators – the score threshold – which is equal to 5 points, and which distinguishes between the high and the low scores. The effectiveness of the measure (marked with *) is indicated by the area bounded by the scores of all its indicators.
CEI data was calculated in Stage 5.

| | **DP (CEI = 2.64*-1.87**-0.96***)** | **SP (CEI = 3.18-1.66-1.20)** | **DC (CEI = 4.81-1.67-1.01)** | **SC (CEI = 4.90-1.75-1.27)** | **NT (CEI = 6.81-1.47-0.01)** | **Evaluation threshold** |
|---|---|---|---|---|---|---|
| Precrop residues, % | 1.06 | 1.16 | 2.67 | 2.53 | 6.11 | 5.00 |
| Earthworms' biomass, g $m^{-2}$ | 1.06 | 2.59 | 3.75 | 4.79 | 7.04 | 5.00 |
| Soil aggregate stability, % | 2.77 | 4.10 | 6.59 | 7.47 | 8.32 | 5.00 |
| Saccharase activity, mg glucoses 1 g of soil per 48 h | 2.69 | 2.56 | 4.70 | 4.66 | 7.93 | 5.00 |
| Crop density before harvest, units $m^{-2}$ | 5.63 | 5.51 | 6.33 | 5.05 | 4.68 | 5.00 |

5) The calculation of the complex evaluation index (CEI), which consists of the average of the evaluation scores (*-EP), the standard deviation of the evaluation scores (**-EP) and the standard deviation of the average of the evaluation scores below the evaluation threshold (***).

CEI and EP calculations were performed by the computer program STAT_ENG in SELEKCIJA software (vers. 5.00, author dr. Pavelas Tarakanovas, Lithuanian Institute of Agriculture, Akademija, Kedainiu distr., Lithuania) was applied. This data was included in the figure legend.

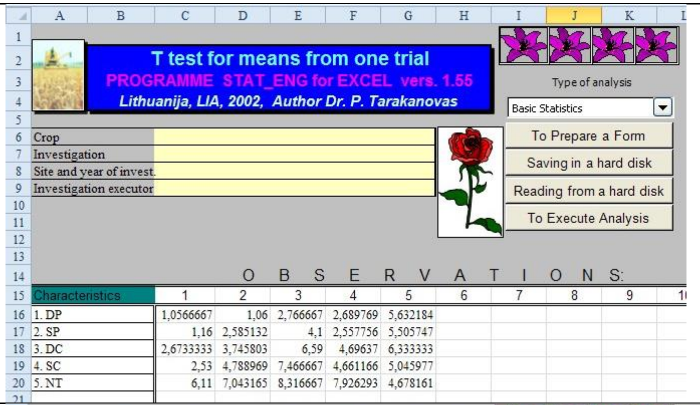


| Characteristics | 1 | 2 | 3 | 4 | 5 | 6 | 7 | 8 | 9 |
|---|---|---|---|---|---|---|---|---|---|
| 1. DP | 1,0566667 | 1,06 | 2,766667 | 2,689769 | 5,632184 | | | | |
| 2. SP | 1,16 | 2,585132 | 4,1 | 2,557756 | 5,505747 | | | | |
| 3. DC | 2,6733333 | 3,745803 | 6,59 | 4,69637 | 6,333333 | | | | |
| 4. SC | 2,53 | 4,788969 | 7,466667 | 4,661166 | 5,045977 | | | | |
| 5. NT | 6,11 | 7,043165 | 8,316667 | 7,926293 | 4,678161 | | | | |

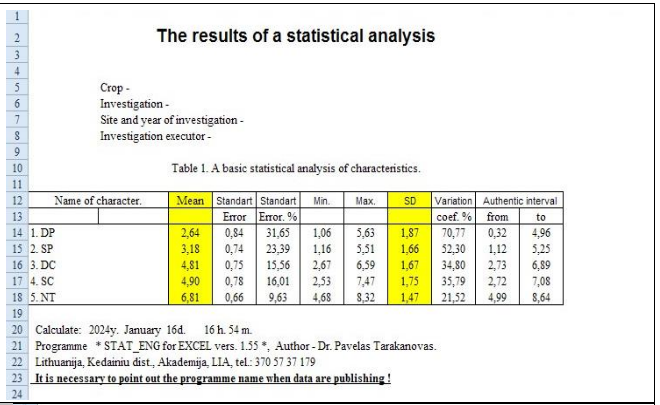
The results of a statistical analysis

Crop -
Investigation -
Site and year of investigation -
Investigation executor -

Table 1. A basic statistical analysis of characteristics.

| Name of character. | Mean | Standart Error | Standart Error. % | Min. | Max. | SD | Variation coef. % | Authentic interval from | to |
|---|---|---|---|---|---|---|---|---|---|
| 1. DP | 2,64 | 0,84 | 31,65 | 1,06 | 5,63 | 1,87 | 70,77 | 0,32 | 4,96 |
| 2. SP | 3,18 | 0,74 | 23,39 | 1,16 | 5,51 | 1,66 | 52,30 | 1,12 | 5,25 |
| 3. DC | 4,81 | 0,75 | 15,56 | 2,67 | 6,59 | 1,67 | 34,80 | 2,73 | 6,89 |
| 4. SC | 4,90 | 0,78 | 16,01 | 2,53 | 7,47 | 1,75 | 35,79 | 2,72 | 7,08 |
| 5. NT | 6,81 | 0,66 | 9,63 | 4,68 | 8,32 | 1,47 | 21,52 | 4,99 | 8,64 |

Calculate: 2024y. January 16d. 16 h. 54 m.
Programme * STAT_ENG for EXCEL vers. 1.55 *, Author - Dr. Pavelas Tarakanovas.
Lithuanija, Kedainiu dist., Akademija, LIA, tel.: 370 57 37 179
**It is necessary to point out the programme name when data are publishing !**

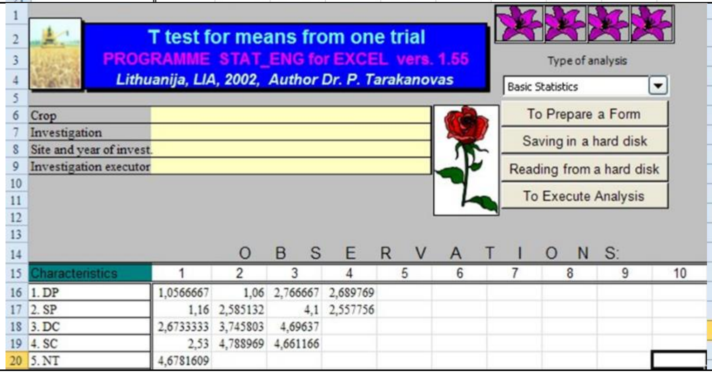


| Characteristics | 1 | 2 | 3 | 4 | 5 | 6 | 7 | 8 | 9 | 10 |
|---|---|---|---|---|---|---|---|---|---|---|
| 1. DP | 1,0566667 | 1,06 | 2,766667 | 2,689769 | | | | | | |
| 2. SP | 1,16 | 2,585132 | 4,1 | 2,557756 | | | | | | |
| 3. DC | 2,6733333 | 3,745803 | 4,69637 | | | | | | | |
| 4. SC | 2,53 | 4,788969 | 4,661166 | | | | | | | |
| 5. NT | 4,6781609 | | | | | | | | | |

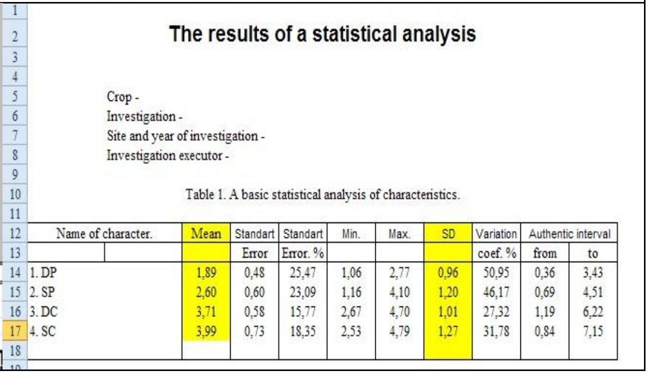
The results of a statistical analysis

Crop -
Investigation -
Site and year of investigation -
Investigation executor -

Table 1. A basic statistical analysis of characteristics.

| Name of character. | Mean | Standart Error | Standart Error. % | Min. | Max. | SD | Variation coef. % | Authentic interval from | to |
|---|---|---|---|---|---|---|---|---|---|
| 1. DP | 1,89 | 0,48 | 25,47 | 1,06 | 2,77 | 0,96 | 50,95 | 0,36 | 3,43 |
| 2. SP | 2,60 | 0,60 | 23,09 | 1,16 | 4,10 | 1,20 | 46,17 | 0,69 | 4,51 |
| 3. DC | 3,71 | 0,58 | 15,77 | 2,67 | 4,70 | 1,01 | 27,32 | 1,19 | 6,22 |
| 4. SC | 3,99 | 0,73 | 18,35 | 2,53 | 4,79 | 1,27 | 31,78 | 0,84 | 7,15 |
